# Anatomical Basis of the Myofascial Trigger Points of the Gluteus Maximus Muscle

**DOI:** 10.1155/2017/4821968

**Published:** 2017-11-16

**Authors:** Flavia Emi Akamatsu, Tatiana Mina Yendo, Ciro Rhode, Ana Maria Itezerote, Flávio Hojaij, Mauro Andrade, Wu Tu Hsing, Alfredo Luiz Jacomo

**Affiliations:** ^1^Department of Surgery, Laboratory of Medical Research (LIM 02), Division of Human Structural Topography, Faculty of Medicine of the University of São Paulo (FMUSP), São Paulo, SP, Brazil; ^2^Department of Surgery Medicine, Laboratory of Medical Research (LIM 02), FMUSP, São Paulo, SP, Brazil; ^3^Department of Pathology, Discipline of Telemedicine, FMUSP, São Paulo, SP, Brazil

## Abstract

Myofascial pain syndrome is characterized by pain and limited range of motion in joints and caused by muscular contracture related to dysfunctional motor end plates and myofascial trigger points (MTrPs). We aimed to observe the anatomical correlation between the clinically described MTrPs and the entry point of the branches of the inferior gluteal nerve into the gluteus maximus muscle. We dissected twenty gluteus maximus muscles from 10 human adult cadavers (5 males and 5 females). We measured the muscles and compiled the distribution of the nerve branches into each of the quadrants of the muscle. Statistical analysis was performed by using Student's* t*-test and Kruskal-Wallis tests. Although no difference was observed either for muscle measurements or for distribution of nerve branching among the subjects, the topography of MTrPs matched the anatomical location of the entry points into the muscle. Thus, anatomical substract of the MTrPs may be useful for a better understanding of the physiopathology of these disorders and provide basis for their surgical and clinical treatment.

## 1. Introduction

Chronic pain is a major public health burden and its reported prevalence ranges widely between 2 and 45% [1].

Musculoskeletal problems account for most cases of chronic pain and myofascial pain syndrome and its prevalence among all causes of this chronic musculoskeletal pain is estimated to be 13,7% to 47% [2–7].

Yet, large population studies are scarce and difficult to be undertaken due to methodological complexity and many studies focus on specific disorders. Also, fibromyalgia (FM), whose reported prevalence is 3–5% of the adult population [8], shares many clinical and physiopathological features with myofascial pain syndrome (MPS).

MPS is clinically expressed by referred pain [7], limited range of motion in joints, and a local twitch response triggered by mechanical stimulation of certain muscular and fascial regions [9]. These focal hypersensitivity areas are known as myofascial trigger points (MTrPs) [2, 8, 10, 11] and are associated with dysfunctional motor endplates [8, 10, 12–14]. Nevertheless, the lack of detailed anatomical information still constitutes a major setback for the complete understanding of physiopathology and larger clinical application of MTrPs.

The gluteus maximus muscle (GMax) is the largest and most superficial muscle of the gluteal region or hip. It is a thick and wide quadrangular muscle with an overlying fascia and molds the gluteal prominence. The muscle has a thick fascicular architecture, with wide bands of fibers separated by fibrous septa. It originates in the posterior gluteal line of the ilium, the iliac crest, and the aponeurosis of the erector spinae and on the dorsal surface of the lower portion of the sacrum and the lateral aspect of the coccyx, the sacrotuberous ligament, and the fascia that covers the gluteus medius muscle. There may be additional bands coming from the lumbar aponeurosis or ischial tuberosity. Also, the muscle can be bimorph. The fibers of the upper portion of the muscle join the superficial fibers of the lower portion to form a thick tendinous lamina that crosses the greater trochanter and inserts into the iliotibial tract of the fascia lata. The deep fibers of the lower portion are inserted in the gluteal tuberosity, between the vastus lateralis and adductor magnus [15].

The upper margin of the gluteus maximus muscle is thin and lies over the gluteus medius muscle. Its prominent lower margin is free and is directed posterolateral and is crossed by the gluteal fold that marks the upper limit of the posterior aspect of the thigh [15].

The gluteus maximus is innervated by the inferior gluteal nerve (L5, S1, and S2). Its blood supply is provided by the inferior gluteal artery. The superior gluteal artery, the first perforating artery of the profunda femoris, the lateral femoral circumflex artery, the lateral sacral artery, and the internal pudendal artery also contribute to muscle irrigation. Usually, most of the vessels and nerves penetrate the deep surface of the muscle in its central portion.

The main action of gluteus maximus muscle is to extend the hip joint between flexed and standing positions and to assist its lateral rotation during climbing stairs; also it steadies thigh and assists in rising from sitting position. It is highly active in the walking cycle [15].

Three different areas in the gluteus maximus muscle were associated with myofascial trigger points [11], but anatomical data concerning these trigger points is still lacking in the literature.

According to Simons, 2005 [11], there are three common sites of trigger points in the gluteus maximus muscle. The first one, MTP1, is adjacent to the sacrum and pain is referred to the sacroiliac joint, the area beside the gluteal cleft, along the gluteal fold, and, occasionally, the posterior aspect of the thigh. Slightly above the ischial tuberosity is MTP2, the most common trigger point of this muscle. It relates to pain in the entire buttock and the lower sacrum and below the crest of the ilium laterally. MTP3 is associated with pain in the coccygeal area and is located within the most medial and inferior muscle fibers.

In the United States the prevalence of hip pain is 14.3% in adults aged 60 years and older [15] and the hip is involved in 2.5% of all sports-related injuries [10]. Gluteus maximus trigger points can be involved in “greater trochanteric pain syndrome” (GTPS), characterized by pain in the region of the greater trochanter [16]. The prevalence of unilateral and bilateral GTPS is 15% [17] and in adults with musculoskeletal low back pain has been reported to be 20% to 35% [18–20].

Our hypothesis was that MTrPs could be related to muscle innervation [21]. The present study's goal is to provide the anatomical localization of the gluteus maximus muscle nerve entry points and its correlation with clinical myofascial trigger points, supplying data that could be useful to better understand its physiopathology and provide anatomical information to be applied in both clinical and surgical settings.

## 2. Materials and Methods

This study was approved by the Ethics Committee of Medical School for the Analysis of Research Projects Protocol 426/12.

Twenty gluteus maximus muscles from 10 human adult cadavers (5 males and 5 females) were dissected to expose the inferior gluteal nerve that innervates the gluteus maximus muscle. The cadavers were previously fixed using a 4% phenolic acid and 0.5% formaldehyde solution and were obtained from a body donation program undertaken by the Discipline of Human Structural Topography of the Department of Surgery of the University, Medical School. Specimens with no sign of previous surgery or any other severe abnormality in the regions of interest were included. The cadavers age ranged from 32 to 92 years (mean 63.3 yrs.). Their approximate heights measured 1.70 to 1.80 m, with an average of 1.72 m. The weight ranged from 42 to 85 Kg (average of 71 Kg). Seven specimens were Caucasian and three had mixed ethnicity.

**Figure 1:**
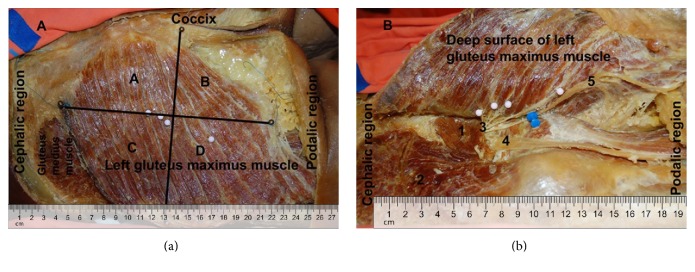
Lateral view of left gluteus maximus muscle. (a) Right gluteus maximus muscle divided into four quadrants: A, B, C, and D. (b) Inferior gluteal nerve entry points on the deep and upper surface of the right gluteus maximus nerve indicated in white pins. 1: piriformis muscle; 2: gluteus medius muscle; 3: inferior gluteal nerve; 4: sciatic nerve; 5: posterior femoral cutaneous nerve.

The specimens were positioned in ventral decubitus on the dissection table and an incision was made at the inferior lumbar region, from the sacral region to the gluteus maximus lateral border. Next, we made an incision straight down along the gluteus maximus lateral border and then along the gluteal sulcus to the medial superior border of the thigh. Flaps of skin and subcutaneous tissue were folded medially to expose the gluteus maximus muscle. We divided and measured the gluteus maximus muscle in four quadrants based on two reference lines: a transversal line through the coccyx and a second line perpendicular to the first, crossing the midpoint between the coccyx and the lateral aspect of the muscle. The quadrant was defined as superomedial (A), superolateral (B), inferomedial (C), and inferolateral (D) (Figure 1(a)).

The deep surface of the muscle was carefully dissected in order to preserve the branches from the inferior gluteal nerve. We observed the inferior gluteal nerve emerging under the inferior border of the piriformis muscle and followed its ramifications to the gluteus maximus muscle. We determined the entry points based on the quadrants previously described (Figures 1(a) and 1(b)).

The statistical analysis was performed by using Student's* t*-test to compare two parametric parameters that have a normal distribution (gluteus maximus dimensions; number of the inferior gluteal nerve entry points into the left and the right gluteus maximus muscle; number of the inferior gluteal nerve entry points into the left and the right female gluteus maximus; number of the inferior gluteal nerve entry points into the left and the right male gluteus maximus; number of the inferior gluteal nerve entry points into the left muscle female and male; number of the inferior gluteal nerve entry points into the right muscle female and male; total number of entry points in the muscle of male and female) and Kruskal-Wallis test to compare multiple groups with a nonparametric parameter difference among the quadrants (A, B, C, and D).

## 3. Results

The transversal measurement of the muscles ranged from 11.6 to 17.0 cm (mean = 14.6 cm). Longitudinal length ranged from 16.0 cm to 20.5 cm (mean = 18.4 cm). The upper portion ranged between 7.5 cm and 13.5 cm, and the lower portion ranged from 4.5 cm to 10.7 cm. There was no statistically significant difference in the morphometric measurements of any quadrant in comparing the right with the left side. However, measurements of transversal and longitudinal segments of the muscle were different in both sides (*p* < 0.0001).

**Figure 2:**
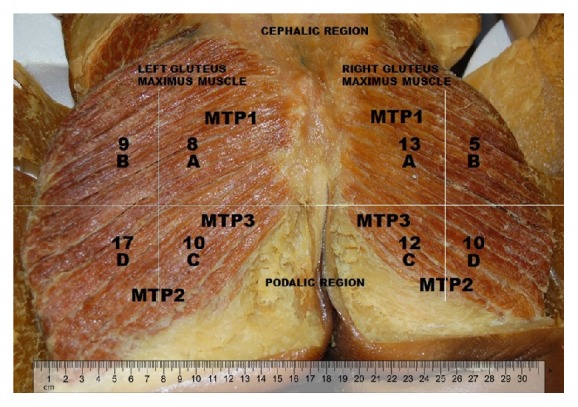
Superficial representation of the entry points of the inferior gluteal nerve into the gluteus maximus muscle. A, B, C, and D represent the quadrants previously designated. The numbers refers to the entry point found in 20 cadavers. MTP refers to myofascial trigger points described by Simons et al., 2005.

In all specimens, the inferior gluteal nerve emerged in the gluteal region after having exited the pelvic region through the lower part of the greater sciatic foramen underneath the piriformis muscle and medial to the sciatic nerve. Then, the nerve branches supply the gluteus maximus through its deeper surface. A communicating branch with the posterior femoral cutaneous nerve may also be present (Figure 1(b)). We found entry points of the inferior gluteal nerve in all quadrants, with a mean of two entry points in A and B and three entry points in C and D quadrants (Figure 2).

We observed that the number of the inferior gluteal nerve entry points into the left and the right gluteus maximus muscle of the same specimen was the same. However, we found that the total number of entry points in the muscle could vary from two to nine. No differences could be attributed to gender.

So, we observed that the distribution of the inferior gluteal nerve entry points into the gluteal maximus muscle was equal in female and male cadavers and also if individual quadrants were considered, the number of entry points was similar.

## 4. Discussion

Nerves are responsible for muscular contraction through liberation of acetylcholine in endplates. Although mechanisms that lead to development of the taut band are not completely elucidated, disorders of the motor endplate are considered to be a probable cause.

Clinically, the myofascial pain results from a motor dysfunction of the muscle characterized by a constant, muscle tenderness that can be felt as a taut band or nodularity within the belly of the muscle. Pain can be local to the site of the taut band and distant from it or referred to another part of the body [22].

Increased concentration of acetylcholine (ACh) in the synaptic cleft, changes in ACh receptor (AChR), and changes in acetylcholinesterase (AChE) activity are consistent with known mechanisms of endplate dysfunction and could explain the increase in endplate electrical activity that is seen in the active MTrPs [22].

The correspondence of the location of the clinically described trigger points of MPS to the topography of the anatomical entry points of the inferior gluteal nerve into the gluteus maximus muscle seems to be a logical explanation for eliciting MTrPs activity.

In our study, we found a perfect anatomical match between the muscle trigger points of the gluteus maximus and the anatomical entries of the branches of the inferior gluteal nerve into the muscle belly. Therefore, comparing our findings to those described by Simons and colleagues [11], we found that MTP1 is located in quadrant A and MTP 2 and MTP 3 are located in quadrants C and D (Figure 2), respectively. We also found nerve entry points in quadrant B, but not in all cadavers. Nevertheless, we may imply that they are the occasional trigger points found along the lateral border of the gluteus maximus or along its attachment to the crest of the ilium, as described by Simon et al., 2005.

Trigger points of the gluteus maximus muscle may also be the cause of either acute or chronic low back pain. A possible way to alleviate symptoms caused by disorders related to MTrPs is by direct approach using acupuncture, dry needling treatment, or other kinds of physical therapies, and clinical improvement has been reported by some authors [22–26].

We would certainly provide conclusive data if we could correlate these findings with patients with previously diagnosed MPS. However, as the cadavers are obtained from donation, we had no access to their clinical file.

We are currently trying to establish similar correlation in other muscles (masseter, deltoid, hallucis abductor, and gluteus medius) to support this hypothesis.

Nonetheless, as the MTrPs have strong anatomical correlation with innervation of the muscle, we believe that the physiopathology of gluteus maximus myofascial pain is related not only to the muscle and its fascia, but more importantly to its innervation. The knowledge of the anatomical basis of MTrP provides a precise location for clinical applications related to certain painful disorders. The selection of the therapeutic approach should be based upon these findings and according to the clinical presentation. Even though direct evaluation of the motor end plates may provide important additional information as to the role of the nerve stimulation in the physiopathology of the myofascial pain, our study focused only on data obtained from anatomical dissection. We believe that further histological and physiological studies of motor end plates will unveil several crucial details which will bring a more complete knowledge of this challenging problem.

## 5. Conclusions

The knowledge of the anatomic localization of the gluteus maximus muscle nerve entry points will help understand the physiopathology and improve treatments in both clinical and surgical contexts, such as myofascial pain syndrome and relation to myofascial trigger points, treatment of chronic pain, and surgical approaches to the hip. The parameters provided in this study may contribute to avoid iatrogenic lesions in surgical approaches and to understand and improve the treatments to myofascial pain syndrome.

## References

[1] Elliott A. M., Smith B. H., Penny K. I., Smith W. C., Chambers W. A. (1999). The epidemiology of chronic pain in the community. *The Lancet*.

[2] Sola A. E., Bonica J. J., Bonica J. J. (1990). Myofascial pain syndromes. The management of pain.

[3] Hong C.-Z. (2000). Myofascial trigger points: pathophysiology and correlation with acupuncture points. *Acupuncture in Medicine*.

[4] White K. P., Harth M. (2001). Classification, epidemiology, and natural history of fibromyalgia. *Current Pain and Headache Reports*.

[5] Huguenin L. K. (2004). Myofascial trigger points: the current evidence. *Physical Therapy in Sport*.

[6] Chen K.-H., Hong C.-Z., Kuo F.-C., Hsu H.-C., Hsieh Y.-L. (2008). Electrophysiologic effects of a therapeutic laser on myofascial trigger spots of rabbit skeletal muscles. *American Journal of Physical Medicine & Rehabilitation*.

[7] Ge H. Y., Nie H., Madeleine P., Danneskiold-Samsøe B., Graven-Nielsen T., Arendt-Nielsen L. (2009). Contribution of the local and referred pain from active myofascial trigger points in fibromyalgia syndrome. *PAIN*.

[8] Gran J. T. (2003). The epidemiology of chronic generalized musculoskeletal pain. *Best Practice & Research Clinical Rheumatology*.

[9] Harden R. N., Bruehl S. P., Gass S., Niemiec C., Barbick B. (2000). Signs and symptoms of the myofascial pain syndrome: a national survey of pain management providers. *The Clinical Journal of Pain*.

[10] Simons D. G. (2001). Do endplate noise and spikes arise from normal motor endplates?. *American Journal of Physical Medicine & Rehabilitation*.

[11] Simons D. G., Travell J. G., Simons L. S. (2005). Terminology of muscle pain disorders. *Myofascial Pain and Dysfunction: The Trigger Point Manual*.

[12] Karakurum B., Karaalin O., Coskun Ö., Dora B., Üçler S., Inan L. E. (2001). The ‘dry-needle technique’: intramuscular stimulation in tension-type headache. *Cephalalgia*.

[13] Simons D. G., Hong C. Z., Simons L. S. (2002). Endplate potentials are common to midfiber myofacial trigger points. *American Journal of Physical Medicine & Rehabilitation*.

[14] Simons D. G. (2004). Review of enigmatic MTrPs as a common cause of enigmatic musculoskeletal pain and dysfunction. *Journal of Electromyography & Kinesiology*.

[15] Stranding S. (2008). *Gray's Anatomy*.

[16] Williams B. S., Cohen S. P. (2009). Greater trochanteric pain syndrome: a review of anatomy, diagnosis and treatment. *Anesthesia & Analgesia*.

[17] Segal N. A., Felson D. T., Torner J. C. (2007). Greater trochanteric pain syndrome: epidemiology and associated factors. *Archives of Physical Medicine and Rehabilitation*.

[18] Swezey R. L. (1976). Pseudo-radiculopathy in subacute trochanteric bursitis of the subgluteus maximus bursa. *Archives of Physical Medicine and Rehabilitation*.

[19] Collèe G., Dijkmans B. A. C., Vandenbroucke J. P., Rozing P. M., Cats A. (1990). A clinical epidemic-logical study in low back pain. Description of two clinical syndromes. *Rheumatology*.

[20] Tortolani P. J., Carbone J. J., Quartararo L. G. (2002). Greater trochanteric pain syndrome in patients referred to orthopedic spine specialists. *The Spine Journal*.

[21] Akamatsu F. E., Ayres B. R., Saleh S. O. (2015). Trigger points: an anatomical substratum. *BioMed Research International*.

[22] Gerwin R. D., Dommerholt J., Shah J. P. (2004). An expansion of Simons' integrated hypothesis of trigger point formation. *Current Pain and Headache Reports*.

[23] Furlan A. D., Van Tudlder M. W., Cherkin D. (2005). Acupuncture and dry needling for low back pain. *The Cochrane Database of Systematic Reviews*.

[24] Zeng Y., Chung J. W.-Y. (2015). Acupuncture for chronic nonspecific low back pain: an overview of systematic reviews. *European Journal of Integrative Medicine*.

[25] Takamoto K., Bito I., Urakawa S. (2015). Effects of compression at myofascial trigger points in patients with acute low back pain: a randomized controlled trial. *European Journal of Pain*.

[26] Lawand P., Lombardi Júnior I., Jones A., Sardim C., Ribeiro L. H., Natour J. (2015). Effect of a muscle stretching program using the global postural reeducation method for patients with chronic low back pain: a randomized controlled trial. *Joint Bone Spine*.

